# Spatial clustering and genetic diversity of *Mycobacterium tuberculosis* isolate among pulmonary tuberculosis suspected patients, Arsi Zone, Ethiopia

**DOI:** 10.1186/s12890-021-01567-7

**Published:** 2021-06-30

**Authors:** Ketema Tafess, Teresa Kisi Beyen, Sisay Girma, Asnakech Girma, Gilman Siu

**Affiliations:** 1 Institute of Pharmaceutical Science, School of Applied Natural Science, Adama Science and Technology University, Adama, Ethiopia; 2 Department of Public Health, College of Health Sciences, Arsi University, Asella, Ethiopia; 3 Department of Clinical Study, College of Veterinary Medicine and Agriculture, Addis Ababa University, P. O. Box 34, Bishoftu, Ethiopia; 4 Disease Prevention, Training and Research Division, Federal Prison General Hospital, Addis Ababa, Ethiopia; 5 Department of Health Technology and Informatics, The Hong Kong Polytechnic University, Hung Hom, Hong Kong

**Keywords:** *Mycobacterium tuberculosis*, Spatial clustering, Genotyping

## Abstract

**Background:**

Tuberculosis remains a serious public health concern globally. The enormous social, economic, and health impacts of the diseases are attributed to the lack of updated data on the prevalence, geospatial distribution, population structures, and genotypic variants of the circulating *M. tuberculosis*.

**Methods:**

Structured questionnaire, mycobacterial culture, and standard 24-Mycobacterial Interspersed Repeated Units-Variable Number Tandem Repeats (MIRU-VNTR) were employed to collect sociodemographic characters, residence linked information, and genotype the isolates. The retrospective discrete Bernoulli model was used to identify the hot spot districts of sputum smear positivity, and Web-based *Miru-VNTRPlus* were used for the identification of lineages and sublineages.

**Results:**

Out of 832 presumptive pulmonary tuberculosis (PTB) suspects, 119 (14.3%) were smear-positive. In the multivariate binary logistic model, PTB suspected patients in the age groups of 7–25 and 25–34 and those from rural residents were 4.53 (AOR = 4.53; 95% CI 2.25–9.13), 3.00 (AOR = 3.00; 95% CI 1.41–6.35) and 1.65 (AOR = 1.65; 95% CI 1.01–2.70) times at higher risk of turning smear-positive. Eleven (47.8%) districts of Arsi Zone were shown to have a high rate of clustering (RR = 2.27; 95% CI 1.62–3.2) of smear-positive PTB. Of 72 isolates queried for the lineage assignment, 59 (81.9%) were classified into the previously known lineages and 13 (18.1%) were not assigned to any known lineages. Overall, 42 (58.3%) belong to *M*. *tuberculosis* lineage 4 (Euro-American), 16 (22.2%) *M*. *tuberculosis* lineage 3 (Delhi/CAS), and 1 (1.4%) *M. tuberculosis* Lineage 1 (Indo-Oceanic/ East Africa Indian). Further classification to the sublineage indicates that the predominant lineage was Delhi/CAS comprising 16 (22.2%) isolates followed by 15 (20.8%) isolates belonging to Haarlem. The remaining isolates were distributed as 13 (18.1%) TUR, 6 (8.3%) LAM, 4 (5.5%) URAL, 4 (4.5%) NEW-1 and 1 (1.4%) EAI.

**Conclusion:**

Our study showed higher smear-positive results among PTB suspected patients and remarkable spatial variation across districts of Arsi Zone in smear-positive PTB. This information together with the genotypic features could be used as input for the efforts of designing control strategies.

**Supplementary Information:**

The online version contains supplementary material available at 10.1186/s12890-021-01567-7.

## Background

Tuberculosis (TB) remains one of the major public health problems with an estimated 10 million new cases and 1.6 million deaths globally [1]. Ethiopia is 7th among the 30 high-burden TB countries with an annual incidence rate of 164 cases/10,000 population. Despite the several efforts and achievements in the control of TB, the disease is among the major public health problems in the country with an estimated mortality of 24 cases/10,000 population. One of the major reasons for the unabated TB challenge is the uneven distribution of the disease across the country [2]. Particularly, the heterogeneous prevalence of smear-positive TB in small geographic areas such as districts substantially affects the transmission dynamics and control efforts.

Previous epidemiological and spatial–temporal studies conducted in various parts of the country showed that there is a significant geographic variation of tuberculosis burden across various administrative units of the countries such as districts and Kebeles [3, 4]. The variation could be associated with differences in the community and individual level risk factors and discrepancies in the implementation of control strategies [5]. Such small-area variations of TB may have important implications for local and national health policy, to target interventions to those areas and communities at the highest risk. However, previous spatial analyses of TB have been generally conducted using routinely collected TB notification data consisting of limited variables. Particularly those studies usually focus on secondary data from health facilities and lack integrating the defining feature of heterogenicity of tuberculosis burden such as bacterial genetic characters.

The geographic variability of the TB burden is associated with host, bacterial and environmental factors [6]. The physiological and medical parameters of the host play a significant role in maintaining the transmission dynamics of TB. For example, smear-positive pulmonary tuberculosis patients transmit *M. tuberculosis* significantly than smear-negative patients [7, 8], playing a pivotal role in the epidemiology and transmission of tuberculosis. That is why smear microscopy remains the backbone to identify the most infectious individuals despite its limited sensitivity [9]. Subsequently, the TB control program places the greatest emphasis on the early diagnosis of smear-positive PTB to ensure prompt initiation of treatment to reduce the transmission of infection.

Bacterial genetics is a powerful and well-studied factor contributing significantly to the uneven distribution of tuberculosis and disease outcomes. *Mycobacterium tuberculosis* complex (MTBC) is considered as clonal bacteria species sharing 99.9% similarity at the nucleotide level. Nonetheless, recent advances in molecular techniques and genome sequencing approaches acknowledged the existence of huge diversity among this clonal species and divided the MTBC into 7 main human-adapted lineages (L1-7) (Lineage 1 (Indo-oceanic), Lineage 2 (East-Asian), Lineage 3 (East-Africa-Indian), Lineage 4 (Euro-American), Lineage 5 (West-African 1/*Mycobacterium africanum*), Lineage 6 (West African 2), Lineage 7 (Woldia/Ethiopia) and animal-adapted lineages [10]. Lineage 2, 3, and 4 are called the modern lineages distributed across the wide geographic area whereas lineage 1, 5, and 6 are called the ancient lineages that are mainly restricted in a certain geographic area. Lineage 7 (recently named Aethiops vetus) is discovered from the central highland of Ethiopia.

*M. tuberculosis* genotyping has several implications as the genetic variation could determine the phenotypic characteristics like disease outcomes. Genotypes of the strains affect the bacterial growth rates and gene expressions, emergence, and distribution of drug resistance, vaccine efficacy, and clinical presentations. The strain type also influences geographic distribution patterns, transmission capacities, and disease-causing capacities. For example, lineage 2 (Beijing) and lineage 4 (Euro–American) are the most widely distributed and virulent than the West African lineages [11]. Further characterization of the lineages to sub-lineage showed the distinct characteristics of the sub-lineages. From Euro-American lineage, the sub-type Haarlem strain shows higher virulence than the geographically restricted lineages [12]. Intriguingly, the characterization of *M. tuberculosis* isolates from different parts of the world like Ethiopia could also support the impact of genetic variation on disease outcomes, and the development and validation of novel diagnostic approaches.

In this study, we aimed to determine the smear positivity, the geospatial clustering, and the genetic diversity of *M. tuberculosis* isolates obtained from Arsi Zone, Ethiopia. Continuous investigation and detailed understanding of the prevalence, geospatial distribution, population structures, and genotypic variants of the *M. tuberculosis* circulating in the areas could ensure the designing and evaluation of universally effective novel control strategies.

## Methods

### Study setting

The study was conducted at the Asella Teaching Hospital, University hospital of Arsi University, situated in Asella Town, South-East of Addis Ababa. The hospital has 297 beds and acts as a medical referral center for a population of 3.5 million inhabitants in the Arsi Zone and its surroundings. The Zone is found in the central part of the Oromiya National Regional State and astronomically lies between 60 45′ N to 58′ N and 38 32′ E to 40 50′ E. Currently, it is divided into 25 districts: Amigna, Aseko, Bale Gasegar, Chole, Digeluna Tijo, Diksis, Dodota, Enkelo Wabe, Gololcha, Guna, Hitosa, Jeju, Limuna Bilbilo, Lude Hitosa, Merti, Munesa, Robe, Seru, Sire, Sherka, Sude, Tena, Tiyo, Ziway Dugda. Provided that Asella Teaching Hospitals is one of the oldest health facilities with better infrastructure and experienced health personnel as well as the only referral hospital in the Zone, most tuberculosis patients particularly referral cases come to the hospital from all districts.

### Samples collection and sputum smear examination

A facility-based study was conducted at Asella Teaching Hospital between February 2016 and August 2016. Before the sputum collection, a pretested structured interviewer-administered questionnaire was used to collect data on socio-demographic, (age, sex, residence, education), districts of the patients (≥ 7 years old), previous contact with TB cases, and facility service-related data from pulmonary tuberculosis suspected patients followed by the collection of sputum samples at a spot and the next day morning for sputum smear examinations. Age of ≥ 7 years old was considered since they could provide sputum sample after consent was obtained from the parent and/or legal guardian.

### Culture and DNA isolation

**Fig. 1:**
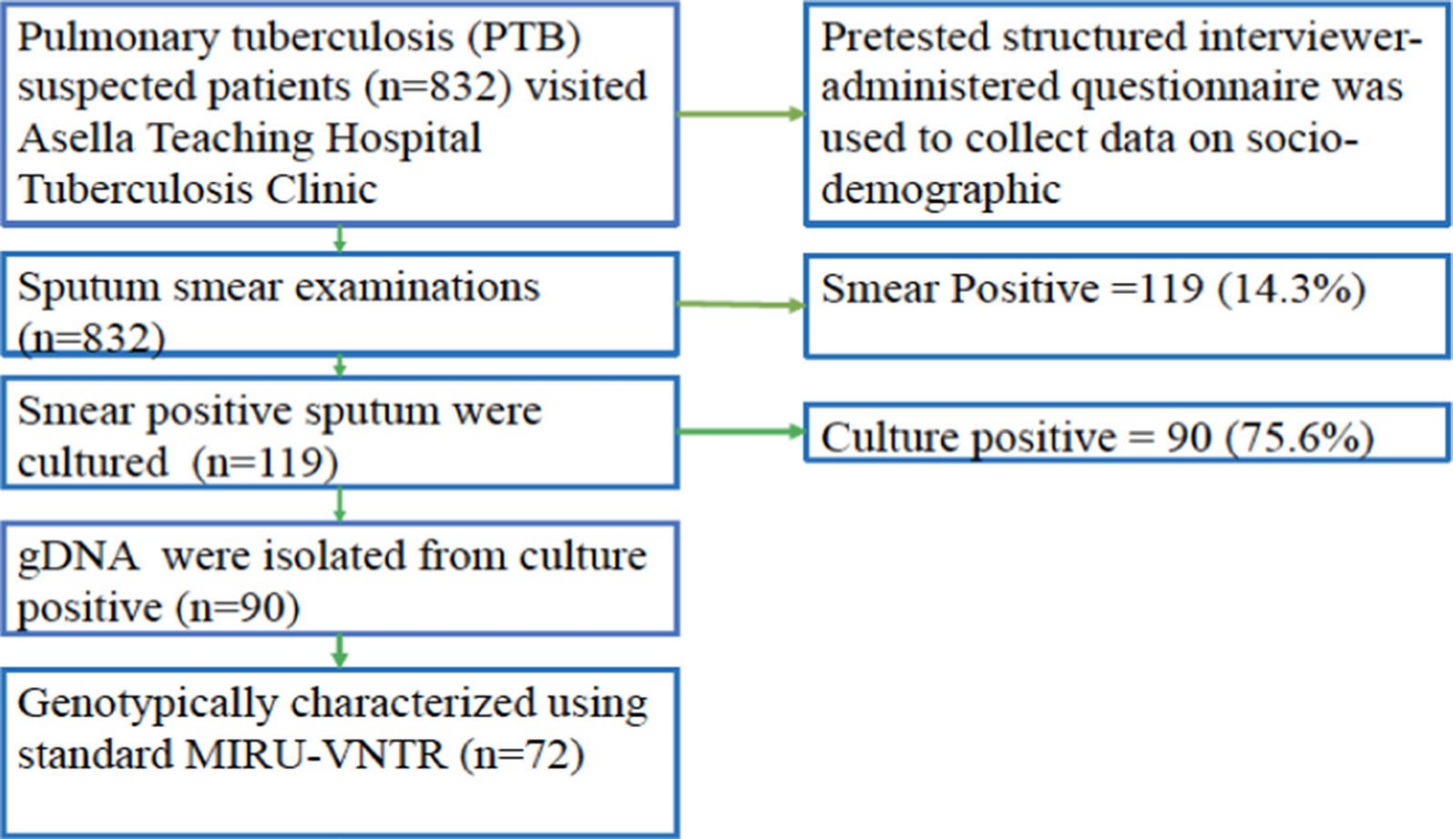
The flowchart demonstrating the number of samples processed at each step during the study

As depicted in Fig. 1, sputum smear positive samples were collected and mycobacterial culture was conducted as described by Forbes et al. [13]. Briefly, the samples were decontaminated and concentrated using N-acetyl L-cysteine (NANC)–NaOH techniques at Adama regional tuberculosis laboratory. The concentrated samples were suspended in 1–2 ml phosphate buffer solution (pH 6.8), thoroughly mixed and 2–3 drops were inoculated into the Lowenstein Jensen (LJ) media and incubated for 4–8 weeks with a regular check-up for the growth. Colonies from culture-positive samples were preserved in 10% glycerol freezing media until required for further processing. Isolation of genomic DNA (gDNA) was conducted on culture-positive isolates using the Roche Cobas Amplicor extraction kit (Roche Diagnostics, USA) as previously described [14]. The DNA samples were shipped to the Laboratory of the Health, Technology and Informatics, The Hong Kong Polytechnic University for genotyping.

### Genotyping using MIRU-VNTR

The PCR amplification of the 24 MIRU-VNTRs loci was conducted using 24 pairs of primers as described previously by Supply et al. [15]. Briefly, amplification was conducted in 96 well plates that enabled the amplification of eight samples per 12 loci. The target loci were amplified in a total of 20 µl mix containing variable proportion of MgCl_2_, 4 µl of Q-solution, 0.08 µl (5 U/µl final concentration) HotStarTaq DNA polymerase, 0.4 µl (10 mM) dNTP, 0.4 µl forward and reverse primers (at 20 µM final concentration), 2 µl 10X PCR buffer, 9.1 µl, and 2 µl of DNA template. The amplification was conducted using Veriti thermal cycler set at initial denaturation at 95 °C for 15 min, 40 cycles of 94 °C for 1 min, annealing at 59 °C for 1 min, extension at 72 °C for 1:30 min, and a final extension of 72 °C for 10 min. The amplified products were mixed with 6X loading dye in a proportion of 5:2 v/v, electrophoresed for 2 h using 1.5% agarose gel (Vivantis Agarose (Molecular Biology Grade) in 1X TBE (Tris–Borate EDTA) buffer with RedSafe™ Nucleic Acid Staining Solution (iNtRON Biotechnology) and visualized under UV transillumination. The product size was compared against the 100 bp ladder and the copy number was determined manually using allele calling tables. Each locus for eight isolates was electrophoresed together to reduces the bias during size determination (Additional file 1: Figure S1).

### Data analysis

After the collected data were entered into the excel spreadsheet, it was exported to SPSS version 22 for descriptive and Binary logistic regression model analysis. For spatial analysis, using the retrospective discrete bernoulli model, the data was exported to SaTScan version 9.6 and Quantum Geographical information system (QGIS) version 3.12. The binary logistic regression model was used to determine the association of patients’ characteristics with sputum smear positivity, while the retrospective discrete Bernoulli model was used to identify the sputum smear positivity hot spot districts. The maximum spatial cluster size was considered as 50% of the population at risk; the Boscoe’s limit on cluster risk level (relative risk) of more than one was used to restrict the high rate cluster. The final significant clusters were declared at a *p* value less than 0.05 (i.e. which combined the Standard Monte Carlo, Sequential Monte Carlo, and Gumbel Approximation) with its 95% CI of relative risks and those which had no Geographical Overlap. The maximum replication was set at 999 of Monte Carlo Replication. Finally, sputum smear-positive hot spot areas were presented by QGIS as per their level of cluster. An analysis of the MIRU-VNTR data was conducted using the browser-based software (http://www.MIRU-VNTRplus.org) as described by Allix-Beguec et al. [16]. The numerical 24 loci copy numbers of isolates were entered into an excel spreadsheet and uploaded. Similarity matching with the standard reference strains was conducted by setting the maximum distance of 0.17 followed by phylogenetic tree classification. Unweighted Pair Group Method with Arithmetic Mean (UPGMA) based dendrogram of the phylogenetic tree was constructed. A cluster rate was calculated as the number of isolates with identical MIRU-VNTR alleles divided by the total number of test isolates. The minimum spanning tree was used to detect the clonal complex of *M. tuberculosis* as well as to assess the genetic links and clonal complexes (CC). The clonal complex was defined as *M. tuberculosis* strains with only two loci differences.

## Results

### Sociodemographic characteristics, prevalence of smear positive and associated factors

A total of 832 PTB suspected patients participated in the study. Out of this, 451 (54.2%) were male and 589 (70.8%) were rural residents. The median age of the patients was 35 years with an interquartile range of 27 and age ranged from 7 up to 86 years. A total of 324 (38.9%) of the patients were illiterate and the majority, 658 (79.1%) had no previous history of hospital admission (Table 1).

**Table 1:** Sociodemographic characteristics and prevalence of smear-positive PTB February 2016, and August 2016, at Asella Hospital, Arsi Zone, Oromia Region, Ethiopia (n = 832)

Characteristics	Total number	Percent (%)	Smear positive (%)		Chi-square	*P* value
			Yes	No		
Sex						
Male	451	54.2	68 (15.1)	383 (84.9)	0.48	0.55
Female	381	45.8	51 (13.4)	330 (86.6)		
Age (years)						
7–25	264	31.7	60 (22.7)	204 (77.3)	28.26	0.000
25–34	148	17.8	24 (16.2)	124 (83.8)		
35–44	139	16.7	13 (9.4)	126 (90.6)		
45–54	101	12.1	9 (8.9)	92 (91.1)		
≥ 55	180	21.6	13 (7.2)	167 (92.8)		
Education						
Illiterate	324	38.9	40 (12.3)	284 (87.7)	2.10	0.55
Elementary	328	39.4	50 (15.2)	278 (84.8)		
High school	122	14.7	21 (17.2)	101 (82.8)		
College and above	58	7.0	8 (13.8)	50 (86.2)		
Residence						
Urban	243	29.2	26 (10.7)	217 (89.3)	3.63	0.06
Rural	589	70.8	93 (15.8)	496 (84.2)		
Previous history of hospital admission						
Yes	174	20.9	23 (13.2)	151 (86.8)	0.21	0.37
No	658	79.1	96 (14.6)	562 (85.4)		
Previous history of imprisonment						
Yes	56	6.7	7 (12.5)	49 (87.5)	0.16	0.43
No	776	93.3	112 (14.4)	664 (85.6)		
The habit of drinking raw milk						
Yes	471	56.6	68 (14.4)	310 (85.6)	0.02	0.49
No	361	43.4	51 (14.1)	403 (85.9		

Out of 832 presumptive PTB patients, 119 (14.3%) were smear-positive with variable tuberculosis prevalence among the studied sociodemographic characteristic. Accordingly, male (15.1%), age group of less than or equal to 25 (22.7%), and rural residents (15.8%) had a higher prevalence of smear-positive PTB. Out of the variables considered in this study, only age groups stand out to be significantly associated with the smear-positive rate (X^2^ = 28.26, *P* = 0.000). Further analysis of the considered variables with the prevalence of smear-positive PTB showed that the age and residence of the patient were the risk factors for the smear positivity. Accordingly, people at age groups of 7–25 and 25–34 were 4.53 (AOR = 4.53; 95% CI 2.25–9.13) and 3.00 (AOR = 3.00; 95% CI 1.41–6.35) times at higher risk of turning smear-positive compared with the old age group (≥ 55 years), respectively. Likewise, though the residence site of the patients was not identified as a risk factor on the bivariate analysis, it becomes a risk factor after adjusting for other confounders on the multivariate analysis (Table 2).

**Table 2:** Crude and adjusted odds ratio for various factors that affect the smear-positive PTB during February 2016, and August 2016, at Asella Hospital, Arsi Zone, Oromia Region, Ethiopia (n = 832)

Character	Smear positive		Crude OD	95% CI	*P* value	Adjusted OD	95% CI	*P* value
	Yes (%)	No (%)						
Sex								
Male	26 (10.7)	217 (89.3)	0.14	0.78–1.70	0.488	1.25	0.82–1.92	0.29
Female	93 (15.8)	496 (84.2)	1			1		
Age (years)								
7–25	60 (22.7)	204 (77.3)	3.78	2.01–7.12	0.000	**4.53**	**2.25–9.13**	**0.00**
25–34	24 (16.2)	124 (83.8)	2.48	1.22–5.01		**3.00**	**1.41–6.35**	
35–44	13 (9.4)	126 (90.6)	1.33	0.59–2.95		**1.47**	**0.64–3.35**	
45–54	9 (8.9)	92 (91.1)	1.26	0.52–3.05		**1.38**	**0.56–3.38**	
≥ 55	13 (7.2)	167 (92.8)	1			**1**		
Education								
Illiterate	40 (12.3)	284 (87.7)	1.23	0.81–1.99	0.553	1.40	0.56–3.47	0.775
Elementary	50 (15.2)	278 (84.8)	1.47	0.83–2.62		1.10	0.47–2.57	
High school	21 (17.2)	101 (82.8)	1.13	0.50–2.57		1.25	0.51–3.11	
College and above	8 (13.8)	50 (86.2)	1		1			
Resident								
Rural	93 (15.8)	496 (84.2)	0.64	0.40–1.01	0.058	**1.65**	**1.01–2.70**	**0.048**
Urban	26 (10.7)	217 (89.3)	1			**1**		
History of hospital admission								
No	96 (14.6)	562 (85.4)	1.121	0.68–1.83	0.64	0.95	0.57–1.59	0.852
Yes	23 (13.2)	151 (86.8)	1			1		
Previous history of imprisonment								
No	112 (14.4)	664 (85.6)	1.18	0.52–2.67	0.69	1.07	0.45–2.53	0.881
Yes	7 (12.5)	49 (87.5)	1			1		
The habit of drinking raw milk								
No	51 (14.1)	403 (85.9	0.97	0.66–1.44	0.89	1.01	0.67–1.51	0.961
Yes	68 (14.4)	310 (85.6)	1			1		

Bold indicate stastically significant variable

### Spatial distribution tuberculosis patients

**Fig. 2:**
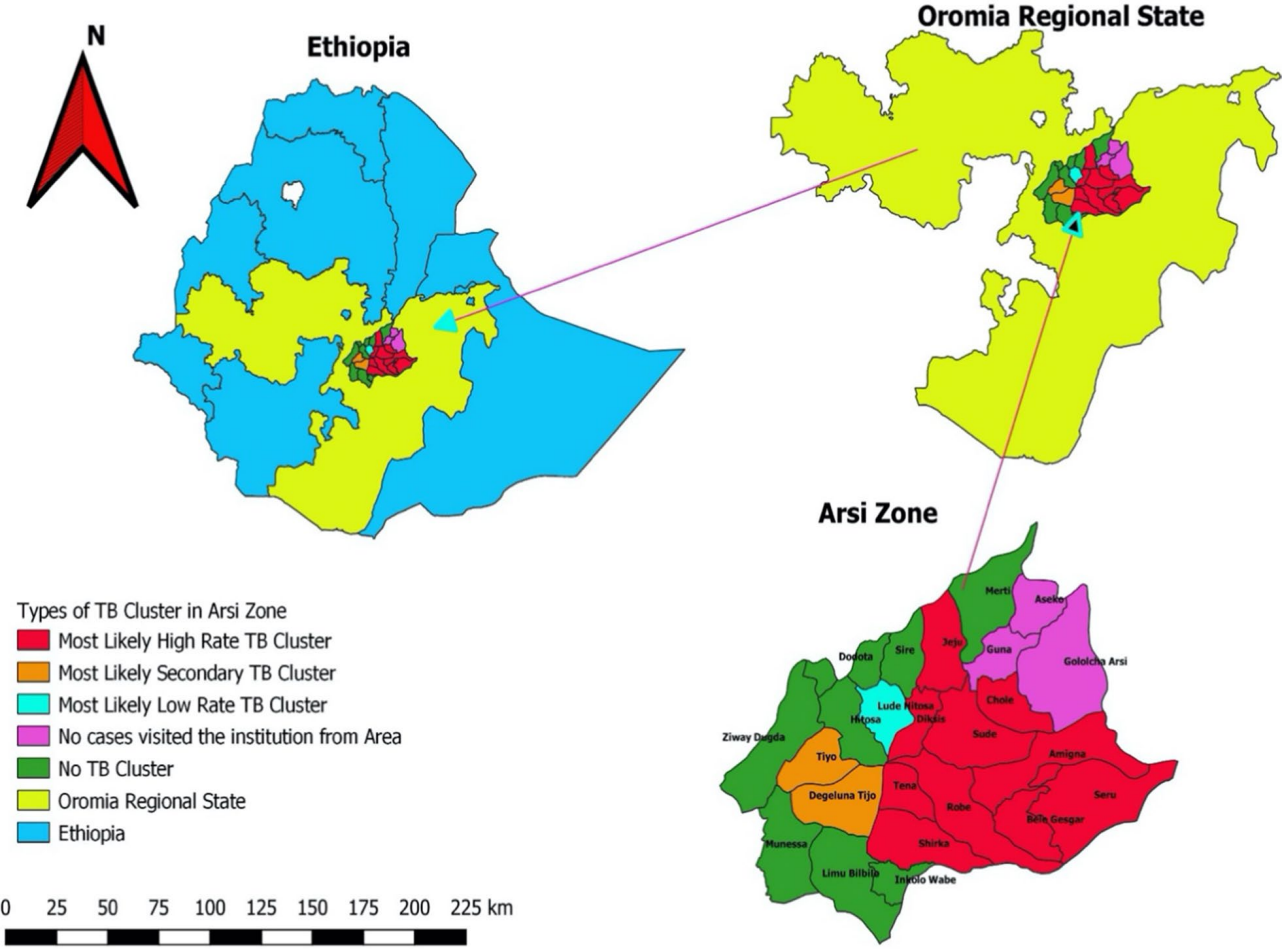
Spatial distribution of smear-positive PTB in the Districts of Arsi Zone, during February 2016, and August 2016, Oromia Region, Ethiopia. The map is generated using the freely accessible Quantum Geographic Information System (QGIS) Developer team (2020). Version 3.12, Open- source Geospatial Foundation Project. http://qgis.osgeo.org”

As illustrated in Fig. 2, presumptive PTB patients visited the hospital from 23 (92%) of the currently 25 districts of Arsi Zone. The disease was significantly clustered in eleven (11) (47.8%) districts (RR = 2.27; 95% CI 1.62–3.2) of the zone while two districts (i.e. Digaluna Tijo, and Tiyo) were identified as potential clusters of the disease (RR = 1.43; 95%CI 0.95–2.16) (Table 3).

**Table 3:** Spatial distribution of smear-positive PTB in districts of Arsi Zone during February 2016, and August 2016, at Asella Hospital, Arsi Zone, Oromia Region, Ethiopia (n = 119)

Cluster type	Lists of districts within the cluster	Number of observed cases	Number of expected cases	RR (95% CI)	Loglikelihood Ratio	*P* value
High rate most likely cluster	Amigna, Shirka, Bale Gesger, Seru, Sude, Chole, Diksis, Jeju, Tena, Robe	38	20.39	2.27 (1.62, 3.2)	9.359710	0.00069
Most likely secondary cluster	Digaluna Tijo, Tiyo	44	36.86	1.43 (0.95, 2.16)	1.450177	0.57
Low rate most likely cluster	Lode Hetossa	1	4.92	0.20 (0.03, 1.37)	2.655211	0.291

### Genetic diversity of *M. tuberculosis*

**Fig. 3:**
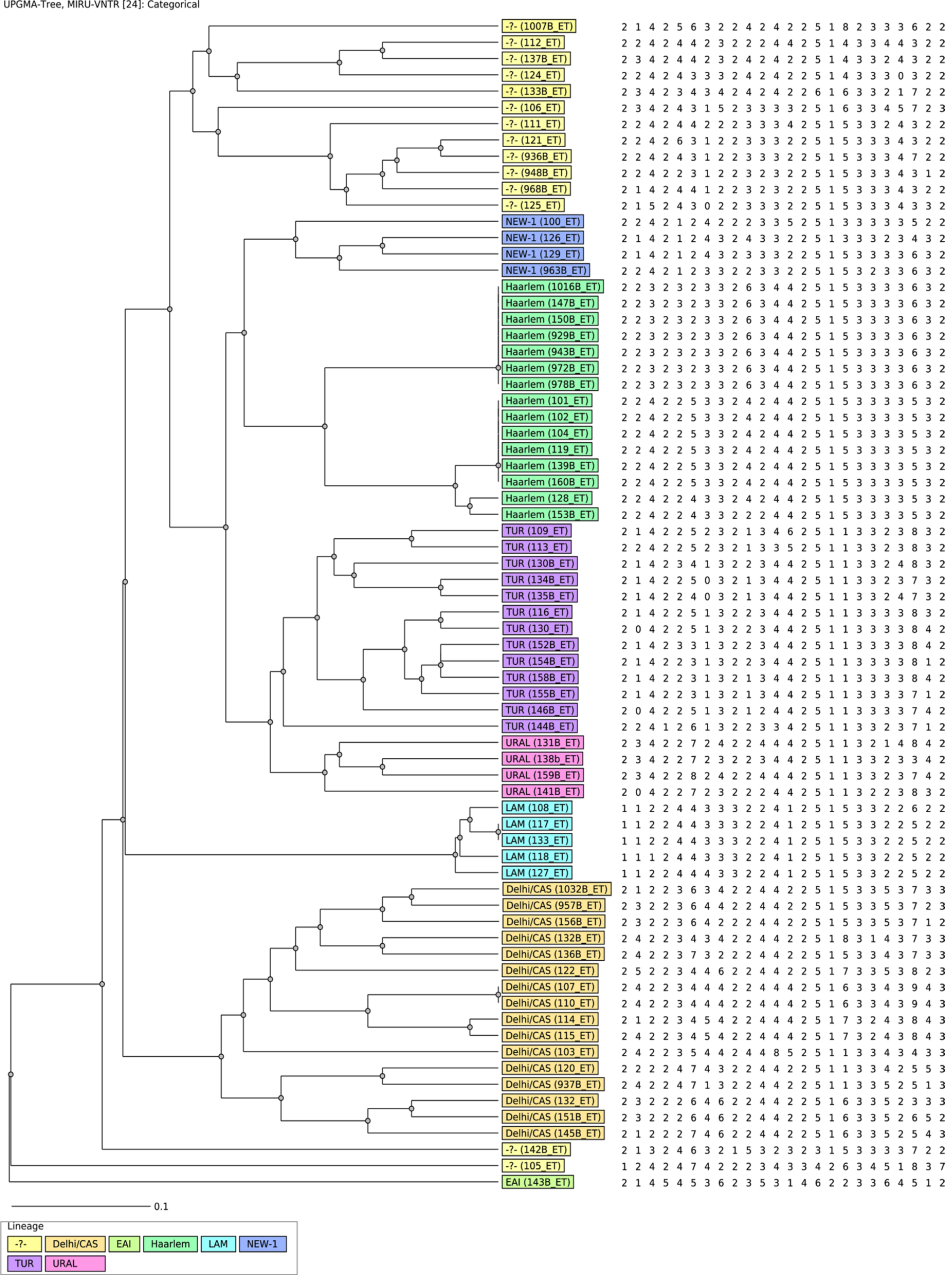
The phylogenetic classification of *M. tuberculosis* isolates isolated from Arsi Zone, during February 2016, and August 2016, Oromia Region, Ethiopia (n = 72)

Out of 90 culture-positive isolates, seventy-two (72 (90%)) isolates were genotypically characterized using standard MIRU-VNTR. Of 72 isolates queried for the lineage assignment, 59 (81.9%) were classified into the previously known lineages and 13 (18.1%) were not assigned to any known lineages. Overall, 42 (58.3%) belong to *M*. *tuberculosis* lineage 4 (Euro-American), 16 (22.2%) *M*. *tuberculosis* lineage 3 (Delhi/CAS), and 1 (1.4%) *M. tuberculosis* Lineage 1 (Indo-Oceanic/East Africa Indian). Further classification to the sublineage indicates that the predominant lineage was Delhi/CAS comprising 16 (22.2%) isolates followed by 15 (20.8%) isolates belonging to Haarlem. The remaining isolates were distributed as 13 (18.1%) TUR, 6 (8.3%) LAM, 4 (5.5%) URAL, 4 (4.5%) NEW-1 and 1 (1.4%) EAI (Table 4). The sub-lineages were distributed across the districts (Additional file 1: Table S1). Further, the comparative results of the study isolate and isolates in the MIRU-VNTR database are provided in Additional file 2: Figure S2. A table comprising information of MIRU-VNTR characterized isolates with country of origin, distrcits of each patient, name and order of each locus is provided as Additional file 3: Table S2 (xls). The constructed phylogenetic tree exhibited a distinct clustering by lineages and sub-lineages as well as isolates were highly diverse and had deep branching (Fig. 3). The analysis of the clustering rate indicated 17 isolates out of 72 (23.6.0%) were grouped into 4 clusters with each cluster composed of 2–7 isolates belonging to the Haarlem sub-lineage. Delhi/CAS and LAM sub-lineages consisting of 1 cluster each composed of 2 isolates.

**Table 4:** The lineage and sub-lineage placement of the *M. tuberculosis* isolates (n = 72)

Lineage	Sub-lineage	Ethiopian isolates (%)
L3 (East-Africa-India)	Delhi/CAS	16 (22.2)
L4 (Euro-American)	Haarlem	15 (20.8)
L4 (Euro-American)	TUR	13 (18.1)
L1 (Indo-oceanic)	EAI	1 (1.4)
L4 (Euro-American)	LAM	6 (8.3)
L4 (Euro-American)	New-1	4 (5.5)
L4 (Euro-American)	URAL	4 (5.5)
–	Not assigned	13 (18.1)
	Total	72 (100)

*L* lineage

### Minimum spanning tree and cluster

**Fig. 4:**
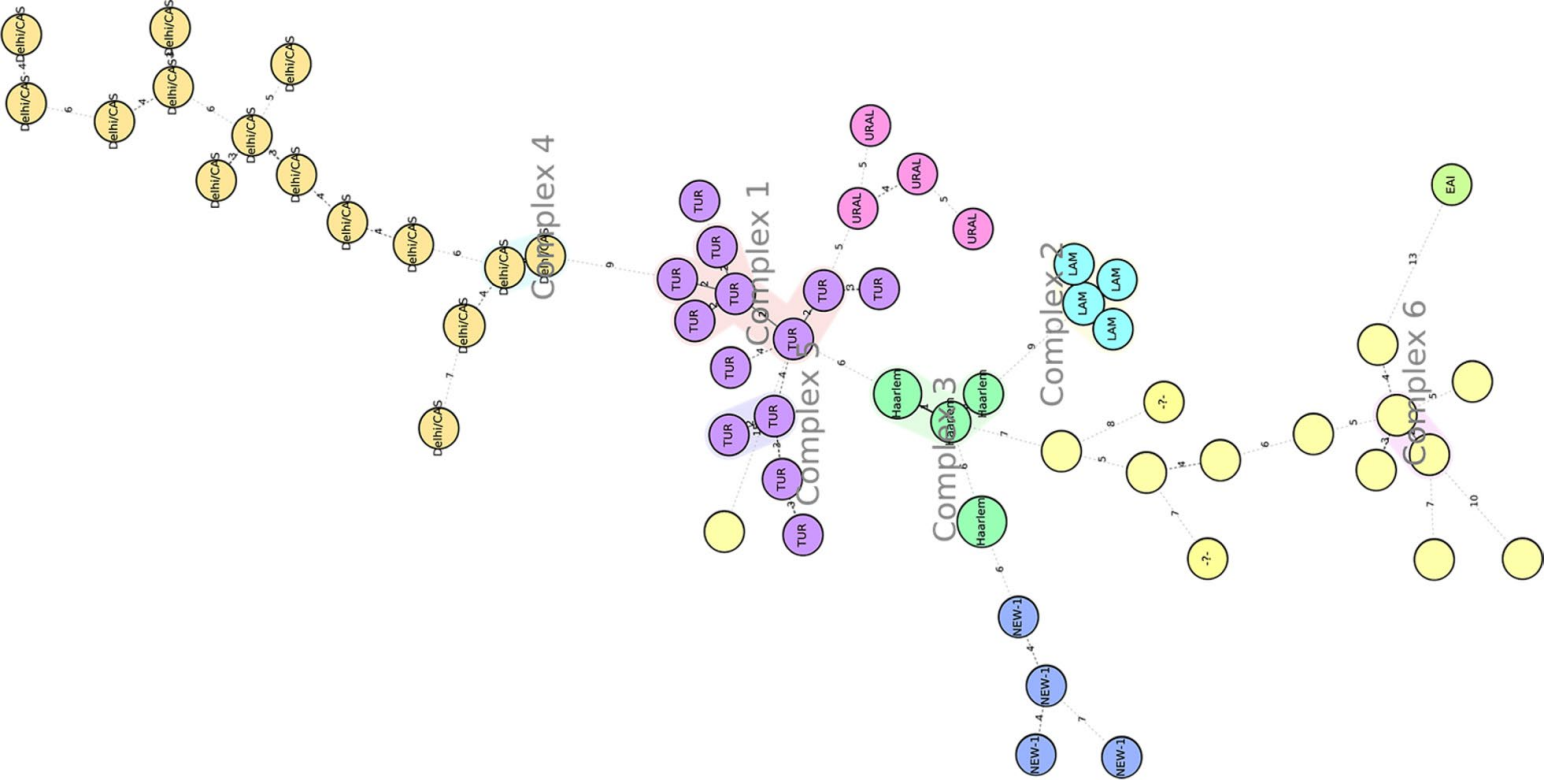
The minimum spanning the tree of the *M. tuberculosis* characterized in this study. The number along the broken line connecting individual circle indicates the genetic distance (number of loci differences) between each strain

The minimum spanning tree (MST) was constructed to identify the clonal complex groups of the isolates within dual locus variants. As shown in Fig. 4, the lineages are indicated by a different color and, the size of the individual circle shows the number of clusters. All the lineages detected using UPGMA analysis were also detected in MST that further confirm genetic linkage and clustering. Six clonal complexes (CC1-6) consisting of a maximum of 6 and a minimum of 2 isolates with dual locus distance were identified. The largest clonal complex (CC1) (5 isolates) belongs to the TUR sub-lineage.

## Discussion

Smear-positive PTB is linked to the higher risk of transmission from an infected individual to a health susceptible individual and hence has of great role in TB epidemiology and infection control. In this study, we investigated the prevalence of smear-positive tuberculosis among presumed PTB suspected patients as well as determined the sociodemographic and geographic risks associated with tuberculosis in Arsi Zone. Further, molecular profiling of the isolates was conducted to identify the circulating strains in the area. Genetic typing enhances the detection of major clones associated with various disease outcomes and mapping out unsuspected outbreaks. Further, characterization is also a foundation for the study of the pathogenesis of the disease that affects virulence, deciphering the MTBC population structure and enabling the development of new diagnostics, vaccines, or treatments.

Out of 832 presumptive PTB suspected patients, 119 (14.3%) were smear-positive. This result is consistent with the 15.2% reported from Bahir Dar, Northwest Ethiopia [17], 14.2% reported from Metehara sugar factory hospital; Central Ethiopia [18], and 16.7% reported from Harar TB control center, Eastern Ethiopia [19]. It is worth noting that the relatively higher smear-positive PTB in the current study and similar results mentioned above are associated with the fact that the participants were presumed tuberculosis patients and/or the cases could be referral cases from the district health facility with a higher likelihood of turning smear-positive. The implication however is; those individuals could be highly infectious and need to be closely monitored to enhance the control of tuberculosis.

The higher risk of smear-positive PTB in young age groups (adolescent and young) compared with the old age in this study is concordant to several previous studies [20–22] and with age-stratified tuberculosis estimates published by the Global Burden of Diseases group [23]. This could be related to the fact that these age groups are at their active period of development and have wider social contact outside their household that could increase the chance of acquiring tuberculosis. Furthermore, many comorbidities relevant to tuberculosis emerge or are exacerbated during these ages including risky substance use (including tobacco use), infection with HIV, and mental health conditions. Consequently, the adolescent and young age groups contribute considerably to the ongoing transmission of tuberculosis particularly in the cases of smear-positive PTB. Provided that these age groups are at higher risk of acquiring tuberculosis necessitates the targeted intervention strategies for effective control of tuberculosis.

After controlling for other factors, the place of residence also was identified as a risk factor in the multivariate analysis. This is contrary to previous studies [24, 25] that identified urban residents as a determinant of the PTB associated with the overcrowded living style in the urban area. On the other hand, the current study is also in agreement with a study from China [26] and a case–control study conducted in Ambo Hospital [27] where being a rural resident is one of the predictors of smear-positive PTB. The discrepancies could be attributed to differences in the target study subject and the lifestyle of the studied population.

Knowledge of the spatial distribution of tuberculosis could augment the control and prevention efforts by identifying high burden areas with low cases detection and weak public health programs. Such identification allows the policymakers to establish priorities for intervention, including TB diagnosis and treatment service to halt the transmission. In this study, significant clustering of smear-positive PTB was identified in 11 (47.8%) districts that cover areas central to remote Eastern parts of the Arsi zone. The results could suggest that sputum smear-positive cases in the study districts were not randomly distributed but in clusters in a spatial pattern. Molecular characterization of the isolates from the clustered districts showed that Delhi/CAS, LAM, and Haarlem are the most frequently identified sublineage (Additional file 1: Table S1). The information could serve as a piece of background information to undertake a large-scale investigation to develop appropriate strategies to improve tuberculosis control schemes.

The majority (71.1%) of the isolates belong to the Euro-American lineage which is consistent with the previous reports [10, 28] suggesting the successful and widespread distribution of this lineage in Ethiopia. Further classification of the Euro-American lineage to sub-lineages indicated the deep-branching of the lineage and the high diversity of the strains belonging to this lineage suggesting the higher *M. tuberculosis* genetic repository potential of the area. We also speculate that diversity could also reflect the potential distance between the patients from whom the samples were collected. *M. tuberculosis* Delhi/CAS and Haarlem represent the largest proportion (accounting for 22.2% and 20.8%), respectively.

A recent systematic review by Tulu and Ameni [29] also indicated the high prevalence of the Delhi/CAS and Haarlem sub-lineage among the diverse isolates and similarly, a study by Tessema et al. [30] also identified *M. tuberculosis* Delhi/CAS and Haarlem as 1st and the 3rd common sub-lineage among the isolates included in their study. Though Delhi/CAS is presumed to be geographically localized in Indian and Central Asia, two assumptions were forwarded for the distribution of this sub-lineage in Ethiopia. The currently growing bidirectional economic and social relationship between Ethiopia and India might have increased the transmission dynamics of these strains from India to Ethiopia. Or East Africa, specifically Ethiopia, is assumed to be the origin of human tuberculosis, thus, strains of *M. tuberculosis* might have migrated to India and central Asia following the “Out of Africa” theory.

Mycobacterium Haarlem sublineage is ubiquitously distributed worldwide, and representing about a quarter of mycobacterial isolates in central America, Europe, and the Caribbean region linked with the European movements during colonization [31]. Studies have also shown that these strains are actively transmitting in an urban setting and responsible for prolonged outbreaks of drug-resistant tuberculosis [32, 33]. Several studies have also proven the widespread distribution of the Haarlem sublineage in various parts of Ethiopia reinforcing our finding [34–36]. The widespread dissemination and clustering of the sublineage in the study area could suggest the high transmission ability and the active transmission of this strain in the population warranting the strengthening of the control efforts to halt the spread.

Though *M. tuberculosis* TUR sub-lineage which is believed to predominant in Turkey and Eastern European countries is occasionally reported from Ethiopia, this is the first report that identified the considerable number (18.1%) of TUR sub-lineage which formed large clonal complexes of 7 isolates in the study area. This might be associated with few studies conducted using MIRU-VNTR based genotyping as most genotyping studies in Ethiopia have been conducted by using a spoligotyping method that classifies TUR sublineage as T1 [37, 38], one of the most prevalent sublineages reported in Ethiopia. Concomitant to our finding recently Wondale et al. [34], using MIRU-VNTR have also identified the TUR sublineage in the South-Omo region of Ethiopia which they reasoned could be linked to the presence of Turkey investors in the South Omo.

The sizeable number of isolates (18.1%) were not assigned to any of the previously known lineage or sublineage in this study. This is consistent with the previous studies in a different part of Ethiopia as reviewed by Tulu et al. [29]. This could support the fact that the East African region in general and Ethiopia in particular, are presumed as the reservoir for the large diversity of MTBC ranging from ancient to new or modern TB lineages. The notion could be supported by the discovery of new mycobacterial lineage in Ethiopia [10] and isolation of the smooth colony tubercle bacilli (*M. canettii*) in East African [39] the species which are considered as the ancestor of MTBC.

Owing to the airborne transmission of *M. tuberculosis*, transmission dynamic estimation using the cluster rate approach would significantly benefit tuberculosis control programs. This would help to identify the factors contributing to the increasing incidence of tuberculosis. Overall, a clustering rate of 26.4% was recorded in this study. The clustering rate for isolates in this study is noticeably lower than previous reports in a different part of the country (reviewed, [29]) which could be related to the discrete nature of the patients from whom the isolates were collected. The Haarlem isolates were identified as the major cluster in Ethiopian isolates suggesting that this strain is responsible for the majority of the recent transmission in the study area.

Our works has also some limitations. We conducted a facility-based study to assess the prevalence of smear positive tuberculosis associated risk factors at Asella Hospital followed by further genetic characterization of the isolates as well as evaluated the geospatial clustering of the case. Thus, it could not be possible to establish the causal relationship between the predictors and the outcome variables. Further, our study only focuses on smear positive pulmonary cases that could limit the evaluation of true burden of tuberculosis in the area.

## Conclusion

Our study showed higher smear-positive results among PTB suspected patients and remarkable spatial variation across districts of Arsi Zone in smear-positive PTB. This information together with the genotypic features could be used as input for the efforts of designing control strategies.

## Supplementary Information

**Additional file 1**.** Figure S1**. The gel-photo of the two loci (MIRU-48 (2461) and MIRU-49 (3171)) and** Table S1**. The distribution of mycobacterial sub-lineages across various districts of Arsi zone.

**Additional file 2**.** Figure S2**. Complementary figure (UPGMA) comparing the isolates of the study and those existing in the MIRU-VNTRplus database.

**Additional file 3**.** Table S2**. Information of MIRU-VNTR characterized isolates with country of origin, distrcits of each patient, name and order of each locus.

## Data Availability

The data used for analysis to support this study are included in the published article.

## Abbreviations

TB: Tuberculosis
PTB: Pulmonary tuberculosis
MIRU-VNTR: Mycobacterial Interspersed Repeated Unit-variable Tandem Repeat
NANC: N-acetyl-L-cysteine
LJ: Lowenstein Jensen
QGIS: Quantum geographical information system
MLS: Minimum spanning tree
CC: Clonal complex
