# Supplementary material for: Spatial clustering and genetic diversity of Mycobacterium tuberculosis isolate among pulmonary tuberculosis suspected patients, Arsi Zone, Ethiopia
Source: BMC Pulm Med. 2021 Jun 30;21:206. doi: 10.1186/s12890-021-01567-7 (PMC8244181; doi:10.1186/s12890-021-01567-7)
Supplement: Supplementary file 1 — Additional file 1. Figure S1. The gel-photo of the two loci (MIRU-48 (2461) and MIRU-49 (3171)) and Table S1. The distribution of mycobacterial sub-lineages across various districts of Arsi zone. [file 12890_2021_1567_MOESM1_ESM.docx]

Spatial clustering and Genetic Diversity of *Mycobacterium Tuberculosis* Isolate among Pulmonary Tuberculosis Suspected Patients, Arsi Zone, Ethiopia.

**Ketema Tafess^1*^, Teresa Kisi Beyen^2^, Sisay Girma^3^, Asnakech Girma^4^, Gilman SIU^5^**

**Author details:**

^1^Department of Medical Laboratory, College of Health Sciences, Arsi University, Asella, Ethiopia

^2^ Department of Public Health, College of Health Sciences, Arsi University, Asella, Ethiopia

^3^Department of Biomedical Science, College of Veterinary Medicine & Agriculture, Addis Ababa University, P.O.Box 34 Bishoftu, Ethiopia.

^4^Disease Prevention and Research Division, Federal Prison General Hospital, Addis Ababa

^5^Department of Health Technology and Informatics, The Hong Kong Polytechnic University, Hong Kong, Hong Kong

**PCR based MIRU-VNTR**

PCR product of each locus was electrophoresed from eight samples per batch to increase the accuracy of recalling copy numbers of each allele. The approach enhanced the detection of the diversity of specific loci among the samples. Distinct bands of the electrophoresed loci were observed for each locus (**Suplementary figure-1)**. The PCR reactions were repeated for the unsuccessful QUB-26 locus by including 1% Dimethyl Sulfoxide **(**DMSO) in the reaction mix.


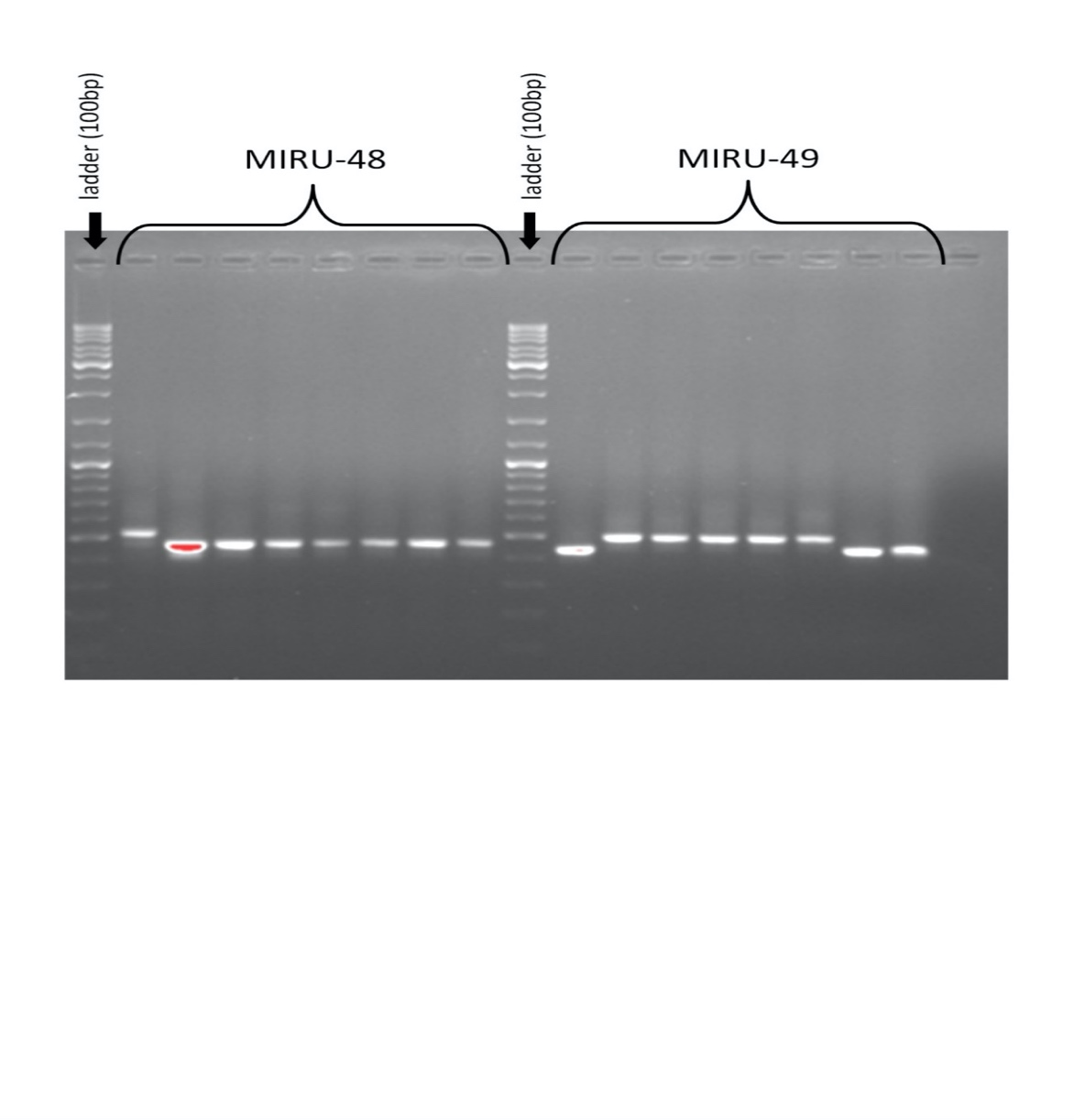


**Figure-S1**: The gel-photo of the two loci (MIRU-48 (2461) and MIRU-49 (3171)) each electrophoresed for 8 samples per batch. The electrophoresis of several samples of the same loci at time enhanced the easy detection of homogeneous and heterogeneous alleles from a different sample.

**Figure S2:**  Complementary figure (UPGMA) comparing the isolates of the study and those existing in the MIRU-VNTRplus database (**Attached on separate page**)

| **Sublineages** | **Woreda/Districts** | | | | | | | | | | | | | **Total** |
| --- | --- | --- | --- | --- | --- | --- | --- | --- | --- | --- | --- | --- | --- | --- |
|  | **Amigna** | **Chole*** | **Degeluna Tijo** | **Diksis*** | **Hetossa** | **Jeju*** | **Limu Bilbilo** | **Munessa** | **Robe*** | **Seru*** | **Shirka*** | **Sire** | **Tiyo** |  |
| Delhi/CAS |  |  | 3 (18.8) | 2 (12.5) | 2 (12.5) |  |  |  | 1 (6.3) | 1 (6.3) | 2 (12.5) |  | 5 (31.3) | 16 (100.  0) |
| EAI |  |  |  |  |  |  |  | 1 (100.0) |  |  |  |  |  | 1 (100.0) |
| Haarmen |  | 1 (6.7) | 3 (20.0) | 1 (6.7) | 3 (20.0) | 1 (6.7) | 1(6.7) | 2 (13.3) |  |  |  |  | 3 (20.0) | 15 (100.0) |
| LAM |  |  |  | 1 (16.7) | 1 (16.7) | 1 (16.7) |  |  | 2 (33.3) |  |  |  | 1 (16.7) | 6 (100.0) |
| NEW-1 |  |  | 2 (50.0) |  |  | 1(25.0) |  |  |  |  |  |  | 1 (25.0) | 4 (100.0) |
| TUR |  |  | 4 (30.8) | 2(15.4) | 2(15.4) |  | 2(15.4) |  |  |  |  | 1 (7.7) | 2 (15.40 | 13 (100.0) |
| URAL |  |  | 2 (50.0) |  | 1(25.0) |  |  |  |  |  |  |  | 1 (25.0) | 4 (100.0) |
| Unknown | 1 (7.7) |  | 2 (15.4) |  | 3 (23.1) |  | 1 (7.7) | 1 (7.7) | 1 (7.7) |  |  |  | 4 (30.8) | 13 (100.0) |
| Total | 1(1.4) | 1 (1.4) | 16 (22.2) | 6 (8.3) | 12 (16.7) | 3 (4.2) | 4 (5.6) | 4 (5.6) | 4 (5.6) | 1 (1.4) | 2 (2.8) | 1 (1.4) | 17 (23.6) | 72 (100.0) |

**Table S1**: The distribution of mycobacterial sub-lineages across various districts of Arsi zone (N=72)

*The marked are districts with the most likely high rates of smear positive TB clusters
